# Notes and News

**Published:** 1897-12-18

**Authors:** 


### Notes and News.

(Collected and Communicated.)

"Lloyd's Newspaper" has collected 1,000 guineas through their penny subscription list for the Prince of Wales's Hospital Fund.

A handsome building has been opened at Bedminster to serve as a branch of the Bristol Dispensary. The cost of building and site was about £2,000.

Dr. Collingridge, the Medical Officer of Health for the Port of London, has been appointed by the Senate of the University of Cambridge one of the examiners for the diploma in public health.

A statue of Pasteur has been erected at Melun, in France, by the Agriculturists and Veterinary Surgeons of the Seine and Marne Department, and a statue to Lavoisier is to be erected in Paris near the Madeleine.

Dr. Collins, the chairman of the County Council, was entertained at dinner on Thursday, December 16th, at the Trocadero Restaurant, by his old fellow-students at St. Bartholomew's Hospital and by other friends.

A new wing—Watford's Jubilee Memorial—has been added to the Cottage Hospital in that town. The main corridor has been extended, and a male ward for six beds has been added. A nurses' room, dispensary, and operating room complete the extensions.

After all, the projected site of the Newcastle Infirmary on the Town Leazes is by no means secured. The last meeting of the City Council ended in a worse state of chaos than ever. It appears that a Bill to hand over the site to the hospital authorities is projected, but first, the very idea of the Bill has met with strong opposition, and secondly, warning is given that the Bill will be opposed if it ever reaches Parliament. In the endeavour to conserve the rights of the freemen their advocates overlook the fact that they are endangering an income of about £3,000 per annum, which the inhabitants of Newcastle will have to find to maintain their hospital, besides the gifts they have already made towards it. If Mr. Hall insists on the establishment of the hospital on the Leazes, and this site is refused, the £100,000 subscribed at the Jubilee will have to be devoted to the building, which will require increased support at the hands of the subscribers.

It is the custom in France to appoint women doctors to girls' *lycées*. Dr. Levraud is now trying to obtain the adoption of the same course in respect to the six professional schools for girls of the city of Paris.

The Assistance Publique of Paris are gradually extending their establishment for incurables at Brévannes, and to it they are now transferring the inmates of the Laennec Hospital in Paris. The home at Brévannes is an old *château* extended, and stands in a beautiful park. It is intended ultimately to provide for upwards of 3,000 inmates. Such a retreat will be an immense boon, serving as it will for a refuge for those cases of phthisis and heart disease, which, while quite unsuitable for the hospitals, require more care than can be bestowed at home.

In the Surbiton Assembly Rooms Sir Squire Bancroft recently gave his famous reading of Charles Dickens's Christmas Carol, in aid of the National Refuges. Commander Hugh H. Hannay, who acted as chairman, passed a well-deserved eulogium upon Sir Squire Bancroft's histrionic abilities and his generosity in thus aiding our principal charities, referred to the National Refuges (on behalf of whose Cottage Hospital at Bisley the reading was given), and made special mention of the society's training ships *Arethusa* and *Chichester*. Over 6,000 lads had passed through these vessels to the Royal Navy and Merchant Service, and in view of the vital importance of supplying British ships with British sailors, such vessels as the *Arethusa* and *Chichester* were supplying a national want.

It is very satisfactory to find that Sidmouth has got over the difficulties which have for some time back stood in the way of the proper disposal of its sewage. In every other respect Sidmouth is so attractive a place that it is only fitting that it should have taken some trouble and spent no inconsiderable a sum of money with this object, and we may fairly congratulate the inhabitants on the completion of the works which have now for some time been in progress. They have been designed by Mr. Mansergh for the repair, and to a large extent the replacement of the main drains, and for the provision of a proper outfall, beyond the town limits, together with the necessary appliances for ensuring the discharge of the sewage at such times of the tide that it will always be carried out of the bay and far away from the sea front of the town. The formal ceremony of declaring the work accomplished was performed by Sir John H. Kennaway, Bart., last week, after which a luncheon was given and many congratulatory speeches were delivered. Sidmouth is one of the warm little nooks that invalids so love. Although, like all English sea-side places, its main season is in the summer, its real therapeutic usefulness comes in as a winter resort for cases of rheumatism, bronchitis, and Bright's disease, and as a refuge for that large number of people who, although they can hardly be called invalids, abhor the cold. Not that it is a stewpan, for on both sides there are high cliffs, and behind it is a great moor on which cool breezes can always be obtained. With its climate, and its fine water supply drawn from a hill some miles away from the town, with its admirable baths, and now with its new drainage, Sidmouth ought to prove very attractive both to healthy people and to invalids.

